# SMARCA4-Deficient Undifferentiated Tumor of the Esophagus: Diagnostic Pitfalls in Immunohistochemical Profiles

**DOI:** 10.1177/10668969241228290

**Published:** 2024-03-18

**Authors:** Rana Chakrabarti, Sherman Lin, Hui Wang, Matthew Cecchini

**Affiliations:** 1 Department of Pathology, 8664 University of Manitoba, Winnipeg, Canada; 2 Department of Pathology, 6221 Western University, London, Canada; 3 Department of Pathology and Laboratory Medicine, 7235 University of Saskatchewan, Saskatoon, Canada; 4 Department of Pathology, 6221 Western University, London, Canada

**Keywords:** SMARCA4, undifferentiated, esophagus, BRG1, immunohistochemistry, pitfalls

## Abstract

SMARCA4-deficient undifferentiated tumors (SMARCA4-UT) are a newly described entity and are typically seen in the thoracic cavity. However, these tumors have been described in other body sites, including the esophagus. These tumors are rare, aggressive neoplasms, characterized by the loss of protein product of SMARCA4 (Brahma-related gene-1) and the preservation of INI1 (SMARCB1) expression. Here, we present two tumors of SMARCA4-UT of the esophagus with its microscopic appearance and immunohistochemical profile. We also include a literature review of SMARCA4-deficient tumors of the tubular gastrointestinal tract with their immunohistochemical and mismatch repair profiles for each specimen. Due to its non-specific histologic appearance and variable staining in expanded immunohistochemical panels, this tumor frequently overlaps with other tumor types, making the diagnosis of SMARCA4-UT challenging. These tumors are often associated with intestinal metaplasia of the esophagus and are thought to represent a high-grade undifferentiated transformation of a conventional esophageal adenocarcinoma. These tumors are typically associated with poor clinical outcomes and have poor response to conventional therapies. Currently, there are no standard guidelines for treatment of these tumors; however, palliative radiotherapy and systemic chemotherapy may provide benefit. More recently, immunotherapy and novel therapeutic targets have shown some promise for these patients.

## Introduction

Switch/sucrose non-fermentable (SWI/SNF) related, matrix associated, actin-dependent regulator of chromatin, subfamily A, member 4 (SMARCA4)-deficient undifferentiated tumors (SMARCA4-UT) are a group of recently characterized entities which includes several tumors arising in the brain, prostate, breast, pancreas, ovary, endometrium, lung, GI, and sinonasal tract.^1^ The vast majority of SMARCA4-UT arise in the thoracic cavity.^2^ These tumors tend to be smoking-related and display strong expression of SOX2, CD34, and SALL4.^3^ Morphologically, these tumors share a number of characteristics including sheet-like proliferation of discohesive cells with focal rhabdoid appearance, large nuclei with prominent nucleoli, and variable necrosis and mitotic activity. While all these lesions share a common underlying abnormality, the pathogenesis of these tumors varies depending on the site. Here, we present two specimens of SMARCA4-deficient undifferentiated carcinomas of the esophagus and their diagnostic challenges.

## Tumor #1

**Figure 1.**
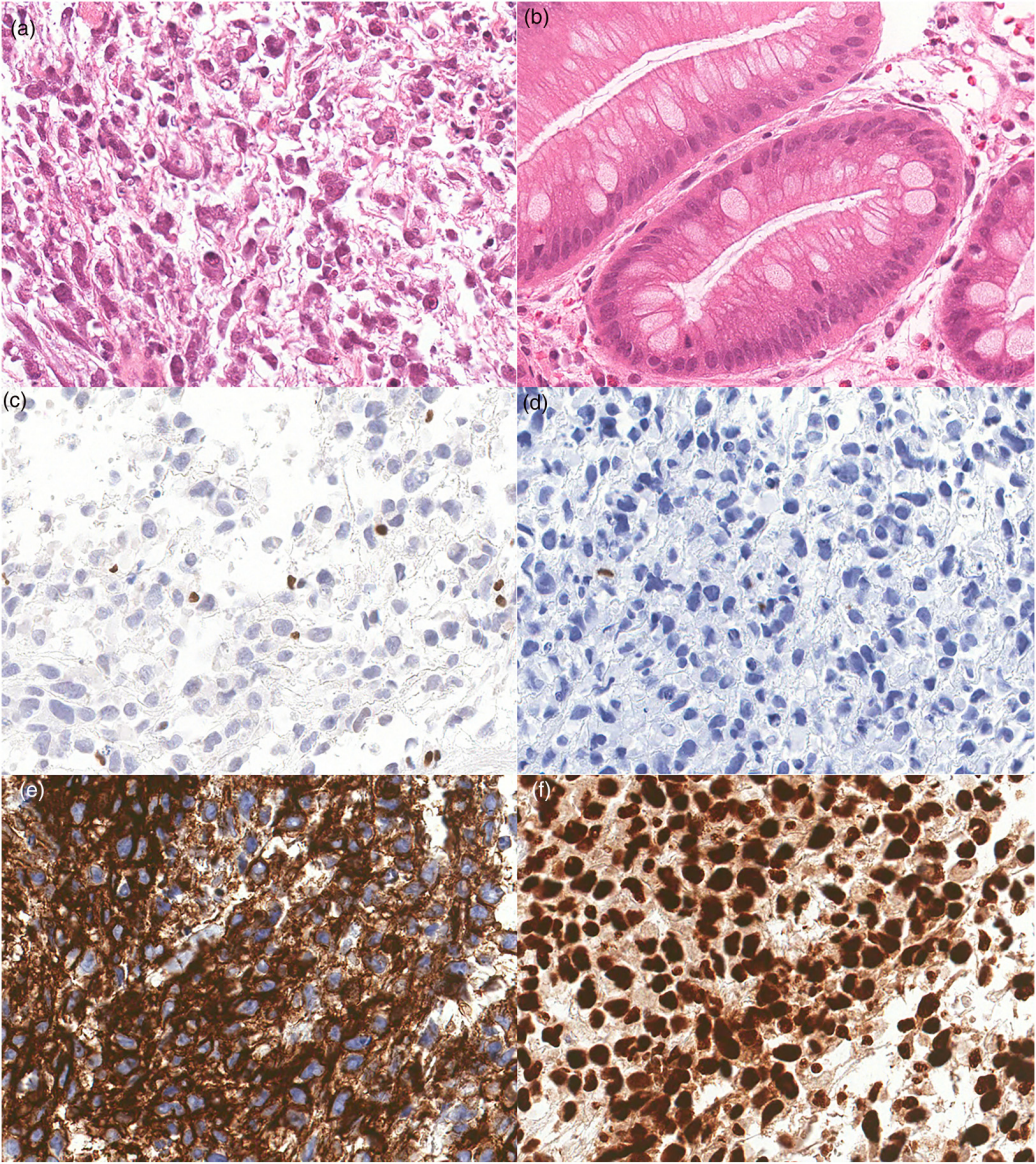
Representative H&E and immunohistochemical staining of SMARCA4-UT. (a) H&E staining of tumor showing rhabdoid morphology and necrosis (40x). (b) H&E staining of background Barrett Esophagus (20x). (c) Tumor cells exhibit loss of protein expression for BRG1 (SMARCA4) (20x). (d) Negative staining in tumor cells for Pan- KRT (20x). (e) Tumor cells stain positively for CD138. (f) Tumor cells show intact expression of INI1 (40x). SMARCA4-UT, SMARCA4-deficient undifferentiated tumors; BRG1, Brahma-related gene-1.

Biopsy of a hemorrhagic, fungating esophageal lesion revealed fragments of a highly necrotic tumor (Figure 1a) in a background of intestinal metaplasia with goblet cells (Figure 1b). The tumor was composed of sheets of discohesive cells with variable nuclear pleomorphism, prominent nucleoli, frequent apoptosis, and focal rhabdoid appearance. Immunohistochemical staining (IHC) was performed to determine lineage, however, tumor cells were negative for pan-keratin (Figure 1d), p40, CDX2, HER2, S100, CD45, SOX10, CD34, synaptophysin, chromogranin A, myogenin, DOG1, KIT (CD117), CD30, and ALK. MLH1, PMS2, MSH2, and MSH6 staining was intact, as was INI1 (SMARCB1) (Figure 1f). No definite mucin staining was identified on a PAS histochemical stain. The tumor cells were positive for CD138 (Figure 1e) and Vimentin. Staining with Brahma-related gene-1 (BRG1), the SMARCA4 protein product, demonstrated loss of expression within tumor cells with intact staining in adjacent stromal cells (Figure 1b). BRG1 staining was also preserved within foci of intestinal metaplasia. The specimen was reported as a SMARCA4-deficient undifferentiated carcinoma of the esophagus.

## Tumor #2

**Figure 2.**
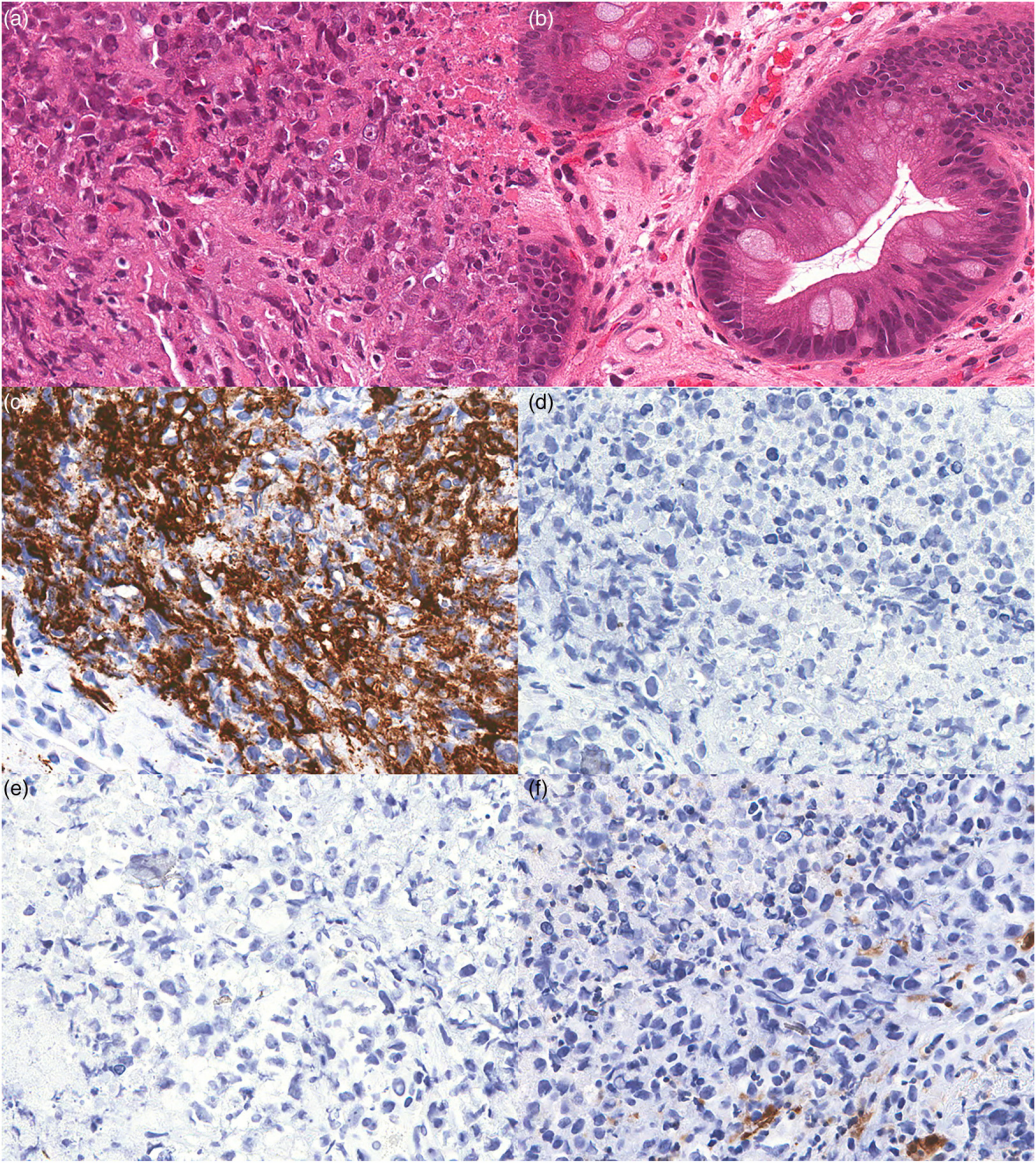
Representative H&E and immunohistochemical staining of SMARCA4-UT. (a) H&E staining of tumor showing rhabdoid morphology and necrosis (40x). (b) H&E staining of background Barrett Esophagus (20x). (c) Tumor cells stain positively for CD34 (20x). (d) Tumor cells exhibit loss of protein expression for BRG1 (20x). (e) p40 (20x), and (f) S100 (20x). SMARCA4-UT, SMARCA4-deficient undifferentiated tumors; BRG1, Brahma-related gene-1.

Endoscopic biopsy of an esophageal mass lesion revealed a necrotic tumor (Figure 2a) arising in a background of intestinal metaplasia (Figure 2b). The tumor cells were arranged in sheets with nuclear pleomorphism, prominent nucleoli, frequent mitoses, and apoptosis with an epithelioid appearance (Figure 2). An extensive IHC panel was performed, and tumor cells were negative for keratin 7, MOC31, CD3, CD20, p40 (Figure 2e), Arginase, S100 (Figure 2f), CD117, DOG1, ERG, CD31, SMA, and CD30. Tumor cells were positive for CD34 (Figure 2c). Subsequent staining for BRG1 was performed which demonstrated loss of protein expression in the tumor cells (Figure 2d). This specimen was also reported as a SMARCA4-deficient undifferentiated esophageal tumor.

## Discussion

SMARCA4 is a critical component of the SWI/SNF complex along with other members including ARID1A, SMARCB1, and SMARCA2. The main role of this complex is to facilitate transcriptional regulation and lineage specification through nucleosome mobilization and chromatin remodeling.^4,5^ In the esophagus, a minority of adenocarcinomas demonstrate loss in one or more components of the SWI/SNF complex. Most frequently, there is the loss of ARID1A (10%), however, the loss of SMARCA2 (9.9%), SMARCA4 (3.4%), and SMARCB1 (2%) is also seen.^5^ Concordant ARID1A and MLH-1 loss has also been reported in 1-2% of cases.^6^

The loss of SMARCA4 alone is insufficient for the classification as a SMARCA4-UT. The diagnosis of SMARCA4-UT requires characteristic morphology and lack of clear differentiation by morphology or immunohistochemistry as described. If such a tumor is suspected, IHC for all the components of the SWI/SNF complex may aid in diagnosis; however at initial diagnosis, a more comprehensive approach should be taken. A literature review of SMARCA4-UT of the tubular gastrointestinal tract demonstrates variable immunohistochemistry and mismatch repair (MMR) staining profiles that makes the diagnosis of this entity challenging (Table 1) and represent potential pitfalls.

**Table 1.** Immunohistochemical Profile of SMARCA4-deficient Tumors of the Tubular Gastrointestinal Tract in the Literature.

Paper	Age	Sex	Site	Positive IHC	Negative IHC	MMR status
Agaimy A. et al, 2016^7^	54	M	Small intestine and ampulla	Pan-KRT Vimentin SMARCA2	KRT7 KRT20 CDX2 P63 ARID1A	MLH1 – Intact PMS2 – Intact MSH2 – Intact MSH6 – Intact
	75	M	Stomach	Pan-KRT KRT7 KRT20 Vimentin SMARCA2		MLH1 – Intact PMS2 – Intact MSH2 – Intact MSH6 – Intact
Agaimy A., et al 2021^8^	66	F	Distal ascending colon	KRT AE1/AE3	SMARCA2 TTF-1 CDX2 SATB2 PAX8 p63 KRT7 KRT20 EMA HepPar-1 Desmin Chromogranin A Synaptophysin CD45 ALK ERG SOX10 Melan-A HMB45	MLH1 – Intact PMS2 – Intact MSH2 – Intact MSH6 – Intact
Chen C. et al, 2021^9^	46	F	Jejunum, Ileum	Pan-KRT KRT7 Vimentin	S100 SOX10 Desmin Myogenin Chromogranin A Synaptophysin CD117 DOG1 KRT20 CDX2 TTF-1 CD24	MLH1 – Intact PMS2 – Intact MSH2 – Intact MSH6 – Intact
Chang B. et al, 2021^10^	74	M	Stomach	INI1 ARID1A EMA Vimentin KRT8 – focal	SMARCA2 Pan-KRT KRT7	Preserved
	60	M	GE Junction	INI1 Vimentin	Pan-KRT EMA KRT8	
	54	M	Small intestine	Pan-KRT – focal SMARCA2 INI1 ARID1A Vimentin	CDX2 KRT20 KRT7	Preserved
	64	M	Colon	INI1 Vimentin	SMARCA2 KRT8 KRT7 KRT20 CDX2	Preserved
	73	M	GE Junction	Vimentin	KRT8 KRT7	
	64	M	Stomach	INI1 Vimentin	Pan-KRT	Preserved
	57	M	Stomach	Pan-KRT Vimentin	KRT7 KRT29 CDX2	
	40	F	GE Junction	INI1 Pan-KRT – focal EMA – focal Vimentin	SMARCA2	
	34	M	Duodenum	INI1 ARID1A Pan-KRT	HER2 KRT7 KRT20 CDX2	Preserved
	58	M	Stomach	INI1	Pan-KRT KRT8 KRT7	
	46	M	Stomach	SMARCA2 INI1 ARID1A Vimentin	Pan-KRT KRT8 KRT7	
	62	M	Duodenum	Vimentin	SMARCA2 Pan-KRT KRT8	
	85	M	GE Junction	INI1 ARID1A	SMARCA2 Pan-KRT	
	72	M	Ileocecal junction	INI1 EMA – focal	SMARCA2 Pan-KRT	
	71	M	Duodenum	Pan-KRT - focal INI1 ARID1A Vimentin	SMARCA2 HER2 EMA KRT8	Preserved
	30	M	Stomach	Pan-KRT - focal INI1 ARID1A Vimentin	SMARCA2 EMA KRT8 HER2	Preserved
	39	F	Colon	INI1 ARID1A Vimentin	SMARCA2 Pan-KRT EMA KRT8 KRT20 CDX2	
	56	M	Duodenum	INI1 ARID1A Vimentin CDX2 – focal	SMARCA2 HER2 Pan-KRT KRT8 KRT7	Preserved
Glückstein MI et al, 2021^11^	-	-	GE Junction	INI1 SMARCA2 EMA KRT7 KRT20 CDX2 p53 – diffuse	Vimentin	
	-	-	GE Junction	INI1 Vimentin EMA	SMARCA2 E-cadherin KRT7 KRT20 CDX2	
	-	-	GE Junction	INI1 SMARCA2 KRT20 CDX2 EMA	KRT7 Vimentin E-cadherin	
Horton R. et al, 2021^12^	63	M	GE junction	OSCAR KRT p53 – diffuse INI1	KRT AE1/AE3 CAM5.2 CDX2 p63 S100 Melan A SMA Desmin CD3 CD20	
	72	M	Esophagus	OSCAR KRT INI1	p63 CD3 CD20	
	77	F	Esophagus	OSCAR KRT INI1 p63	CDX2 p5 – total loss	
	72	F	Esophagus	OSCAR KRT INI1 p53 – diffuse	KRT AE1/AE3 CAM5.2 CDX2 S100 Melan A HMB45 CD3 CD20	
	70	M	Esophagus	CAM 5.2 CDX2	KRT AE1/AE3 SMARCA2 p63 S100 HMB45 SMA Desmin CD3 CD20	
	64	M	Esophagus	KRT AE1/AE3 p63	SMARCA2 S100 Melan A CD3 CD20	
	68	F	GE Junction		KRT AE1/AE3 CAM5.2 SMARCA2 S100 HMB45 CD3 CD20	
	76	M	Esophagus	KRT AE1/AE3 CAM5.2 OSCAR KRT INI1 CDX2	S100 Melan A HMB45 Desmin CD3 CD20	
	63	M	Esophagus		CAM 5.2 SMARCA2	
	79	F	Esophagus	KRT AE1/AE3 INI1	CDX2 S100 HMB45 Desmin	
	77	M	Esophagus	KRT AE1/AE3 CAM5.2 CDX2	SMARCA2 S100 Melan A CD3 CD20	
	73	M	Esophagus	INI1 p53 – diffuse	OSCAR KRT CDX2 S100 Desmin CD3 CD20	
Duan. et al, 2021^13^	61	M	Colon	INI1 KRT8 KRT18 p53 FLI1 SALL4 Vimentin	KRT20 CDX2 Desmin Myoglobin MyoD1 Myogenin CD56 Synaptophysin S100	
Kilic A. et al, 2019^14^	70	M	Stomach	CD138 PAX5	KRT AE1/AE3 CD45 S100 CD99 Desmin OSCAR KRT KRT7 KRT20 EMA TTF-1 PAX8 CDX2 GATA3 CD3 CD20 CD30 CD43 CD79a OCT2 MUM1 ALK Lysozyme Myeloperoxidase Chromogranin A Synaptophysin CD56 CD34 CD117 SOX10 HHV8	
Liu J., et al 2020^2^	40	F	GE Junction	KRT AE1/AE3 INI1 SOX2 CD34	SMARCA2 Claudin-4 CD117 DOG1 α-SMA Desmin CD31 ERG S100	
Nagano H. et al, 2019^15^	67	M	Esophagus – SMARCA4 IHC not done	Vimentin CD34 SMARCB1	KRT AE1/AE3 CAM 5.2 34BE12 KRT5/6 KRT20 Myoglobin CD30 CD45 EMA CA19-9 S100 c-kit LMP-1 Chromogranin A Synaptophysin	
Ota T. et al, 2022^16^	59	M	Stomach	INI1 Vimentin SOX2 SALL4	KRT AE1/AE3 CD45 CD10 CD5 BCL2 CD117 CD34 S100 Desmin MNF116 CD20 CD79a CD3 CD99 NKX2.2 TLE-1 WT1 SOX10 HMB45 α-SMA Claudin-4 NUT CD34	
Tessier-Cloutier B et al, 2020^17^	79	M	Stomach	INI1 ARID1A ARID1B	SMARCA2	PMS2 – Intact MSH6 – Intact
	61	-	Colon	INI1 ARID1A ARID1B		MLH1 – Lost PMS2 – Lost MSH2 – Intact MSH6 – Intact

IHC, immunohistochemical staining; MMR, mismatch repair.

These tumors typically have a relatively characteristic appearance with discohesive cells that have a rhabdoid/plasmacytoid appearance. However, this is not completely specific and other tumors can have an overlapping morphology. Based on morphology, the differential is typically broad and includes poorly differentiated carcinomas, melanoma, undifferentiated sarcomas, and hematolymphoid neoplasms. While there are a number of immunohistochemical stains that are more frequently positive in this lesion (Supplemental Table 2), as with any tumor with such a broad differential diagnosis, it is typically best to start with a small immunopanel that covers major lineages such as Pan- KRT, CD45, and S100.

SMARCA4-UT tend to display little differentiation by immunohistochemistry and routine screening panels highlighted above are often negative (Table 1). This prompts the need for larger immunopanels in an effort to demonstrate lines of differentiation. Focal and sometimes diffuse Keratin AE1/AE3 and CAM5.2 staining may be present and this can be very helpful to support this diagnosis and support the fact that this a poorly differentiated carcinoma.^2,15^ A subset of these tumors can also have focal staining of synaptophysin, chromogranin A, and CD56, which is a diagnostic pitfall that may result in the misclassification of the tumor as a high-grade neuroendocrine carcinoma.^15^ CD138 and PAX5 staining has been reported in a SMARCA4-deficient tumor at the gastroesophageal junction (GEJ)^14^ and is also a pitfall that could result in a misclassification as hematolymphoid neoplasm. CD34 staining may be variable, however, diffuse positive staining of an undifferentiated GEJ tumor can be seen and can also result in misclassification.^2^ Notably, SALL4 staining is also often seen, which can result in the erroneous classification as a germ cell tumor.^13,16^ Given the undifferentiated nature of the tumor and the variable expression of some lineage markers, it is likely in the past that these tumors have been misclassified as hematolymphoid tumors, sarcomas, poorly differentiated carcinomas, vascular tumors, and germ cell tumors.

Similar to our specimen, MMR abnormalities and microsatellite instability-high (MSI-H) are not commonly seen in the esophagus. MMR loss by IHC has been reported in 3-6.6% of esophageal adenocarcinomas^18^ and in a separate study determined that the MSI-H rate in esophageal squamous cell carcinoma and adenocarcinomas is 0.6%.^19^ The rate of MMR loss in SMARCA4 undifferentiated carcinomas of the esophagus has not been characterized; however, elsewhere in the GI system, case reports of MMR loss in SMARCA4-deficient carcinomas have been demonstrated. Tessier-Cloutier et al reported an undifferentiated colonic carcinoma with SMARCA4, MLH1, and PMS2 loss.^17^ There is great variability in MMR status among SMARCA4-deficient carcinomas in the GI tract with reports of tumors in the small intestine and stomach.^9^ An analysis of the TCGA Pan-Cancer Atlas showed 14.7% of any gastric cancer with any SWI/SNF alteration had MSI.^20^ Therefore, it appears that MMR deficiency is not mutually exclusive with SMARCA4 loss and should not preclude the classification as a SMARCA4-UT.

Barrett esophagus are present in both specimens. Though adenocarcinomas of esophagus and GE junction are frequently associated with Barret esophagus in the western world, the relationship of Barrett esophagus and the development of SMARCA4-UT is still unknown. The location of the tumors and the presence of intestinal metaplasia with goblet cells suggest the carcinoma lineage of the tumor which is in line with prior results demonstrating concurrence of Barrett esophagus with this lesion.^12^ Moreover, further testing of BRG1 loss supports the diagnosis of SMARCA4-UT.

There is no standard treatment protocol in place for these patients, and in general, the prognosis for this entity is very poor. SMARCA4-UT carcinomas at extra-GI have showed inadequate response to systemic chemotherapy (albeit with limited short-term benefit with adriamycin and ifosfamide^21^) and surgery^22^ and variable response to palliative radiation therapy.^23^ Patients with chemotherapy-resistant SMARCA4-UT have also shown some benefit using immune checkpoint inhibitors (ICIs) such as pembrolizumab and nivolumab and platinum-based chemotherapy.^22,24^ Due to growing body of literature suggesting the role of tumor mutation burden in the pathogenesis of SMARCA4-UT, the use of ICIs as first line-therapy in these patients is currently being evaluated.^24^ More recently, clinical trials are underway and have shown some promise using novel therapeutic agents including BETi (bromodomain and extra-terminal motif protein inhibitors), enhancer of zeste homolog 2 (EZH2) (NCT03213665; NCT02875548; NCT02601950), cyclin-dependent kinase (CDK)4/6 inhibitors, and histone deacetylase inhibition.^5,21,22,24–26^ Therefore, it is important for pathologists to recognize SMARCA4 loss in poorly differentiated carcinomas both to direct patients towards the most effective existing therapies as well as allowing for patients the opportunity to be enrolled in existing clinical trials.

## Conclusion

SMARCA4-UT present a unique diagnostic challenge for pathologists. While there are common morphologic features, the variability in histology, and IHC staining can lead to improper or delayed diagnoses. Recognizing this diagnostic entity earlier in the diagnostic work up can allow for more targeted and efficient IHC panels and limit the risk of misclassification. SMARCA4-UT are aggressive tumors with a poor clinical outcome, however, there are some emerging therapies which may hold promise in treating these tumors.

## Supplemental Material

sj-docx-1-ijs-10.1177_10668969241228290 - Supplemental material for SMARCA4-Deficient Undifferentiated Tumor of the Esophagus: Diagnostic Pitfalls in Immunohistochemical Profiles Supplemental material, sj-docx-1-ijs-10.1177_10668969241228290 for SMARCA4-Deficient Undifferentiated Tumor of the Esophagus: Diagnostic Pitfalls in Immunohistochemical Profiles by Rana Chakrabarti, Sherman Lin, Hui Wang and Matthew Cecchini in International Journal of Surgical Pathology
