# Supplementary material for: SMARCA4-Deficient Undifferentiated Tumor of the Esophagus: Diagnostic Pitfalls in Immunohistochemical Profiles
Source: Int J Surg Pathol. 2024 Mar 18;32(7):1292–302. doi: 10.1177/10668969241228290 (PMC11440787; doi:10.1177/10668969241228290)
Supplement: sj-docx-1-ijs-10.1177_10668969241228290 - Supplemental material for SMARCA4-Deficient Undifferentiated Tumor of the Esophagus: Diagnostic Pitfalls in Immunohistochemical Profiles [file sj-docx-1-ijs-10.1177_10668969241228290.docx]

**Supplemental Table 2. Frequency of Positive Immunohistochemical Staining of SMARCA4-deficient Tumors of the Tubular Gastrointestinal Tract in the Literature**

| **IHC** | **% Positive** | **Count** |
| --- | --- | --- |
| ARID1B | 100 | 2/2 |
| CD138 | 100 | 1/1 |
| KRT18 | 100 | 1/1 |
| FLI1 | 100 | 1/1 |
| INI-1 | 100 | 30/30 |
| p53 (any) | 100 | 1/1 |
| p53 (overexpression) | 100 | 4/4 |
| PAX5 | 100 | 1/1 |
| SALL4 | 100 | 2/2 |
| SMARCB1 | 100 | 1/1 |
| SOX2 | 100 | 2/2 |
| ARID1A | 92 | 11/12 |
| Vimentin | 91 | 21/23 |
| OSCAR KRT | 71 | 5/7 |
| EMA (any) | 50 | 6/12 |
| CD34 | 50 | 2/4 |
| Pan-KRT (any) | 47 | 15/32 |
| CAM 5.2 | 37.5 | 3/8 |
| EMA | 36 | 4/11 |
| p63 | 29 | 2/7 |
| CDX2 (any) | 27 | 6/22 |
| SMARCA2 | 24 | 6/25 |
| KRT20 | 21 | 3/14 |
| KRT7 | 18 | 3/17 |
| KRT8 (any) | 17 | 2/12 |
